# Air Pollution Exposure and Cognitive Function in Taiwanese Older Adults: A Repeated Measurement Study

**DOI:** 10.3390/ijerph16162976

**Published:** 2019-08-19

**Authors:** Yuan-Ting C. Lo, Ya-Chi Lu, Yu-Hung Chang, Senyeong Kao, Han-Bin Huang

**Affiliations:** 1 School of Public Health, National Defense Medical Center, No.161, Sec. 6, Minquan E. Rd., Neihu Dist., Taipei City 11490, Taiwan; 2 Department of Public Health, China Medical University, Taichung, No.91 Hsueh-Shih Road, Taichung 40402, Taiwan

**Keywords:** air pollution, older adults, cognitive function, neurocognitive disorders

## Abstract

Studies related to air pollution exposure and neurocognitive disorders, specifically cognitive impairment, among older adults are limited. We investigated the association between short-term and long-term exposure to ambient air pollution (i.e., particulate matter with an aerodynamic diameter of <10 μm and ozone) and the effects of their interaction on cognitive function in a community-dwelling, free-living elderly population. Study participants were in a multiple-wave representative sample, namely the Taiwan Longitudinal Study on Aging (n = 2241). In four surveys between 1996 and 2007, their cognitive function was assessed using the Short Portable Mental Status Questionnaire (SPMSQ). We estimated air pollution from 1993 to 2007, including daily concentrations of PM_10_ and O_3_ from air quality monitoring stations, based on the administrative zone of each participant’s residence. Generalized linear mixed models were used to examine these associations after adjusting for covariates. We found that long-term exposure to PM_10_ and O_3_ was significantly associated with cognitive impairment (OR = 1.094, 95% CI: 1.020, 1.174 for PM_10_; OR = 1.878, 95% CI: 1.363, 2.560 for O_3_). The joint effect of exposure to PM_10_ and O_3_ was associated with cognitive impairment (*p* < 0.001). Co-exposure to ambient PM_10_ and O_3_ may deteriorate cognitive function in older adults.

## 1. Introduction

The world’s population is rapidly aging, and the proportion of older adults in the world is estimated to almost double from approximately 12% to 22% [1,2]. In Taiwan, the aging population percentage is likely to reach 39.3% by 2060; moreover, the aging population percentage will be the second highest in the world [3]. More than 20% of adults aged 60 years and more have a neurological disorder [2]. Neurocognitive disorders, including dementia and cognitive impairment, are public health challenges worldwide [1,2,4].

Atmospheric pollution could have deleterious effects on the central nervous system (CNS). Among the various types of pollution, ozone and particulate matter appear to be the most widespread and harmful airborne pollutants [5]. In vivo and in vitro studies have demonstrated that air pollutants, such as particulate matter (PM) and O_3_, are neurotoxicants that can result in neurodevelopmental and neurological damage through inflammation and oxidative stress [6,7,8]. Experimental studies have reported that exposure to a mixture of PM and O_3_ can accelerate brain aging in dogs [9,10]. In particular, ozone may exacerbate the toxicity of other air pollutants like particulate matter by potentiating oxidative stress and inflammation and so consecutive tissue damage, and by contributing to the alteration of the barriers that normally prevent or limit the entry of these pollutants into the brain [5].

Previous longitudinal studies in elderly adults have found associations between certain types of air pollution and neurocognitive disorders [11,12,13,14]. Several studies have not observed any association between exposure to PM_10_ or O_3_ and cognitive decline [15,16,17]. There is still insufficient evidence regarding which pollutant is more relevant and what magnitude of interaction exists between exposure to different air pollutants (e.g., PM and O_3_) and cognitive function. Thus, we used data from the Taiwan Longitudinal Study on Aging (TLSA) to examine the relationships between exposure to PM with an aerodynamic diameter of <10 μm (PM_10_) and O_3_, and the effects of their interaction on cognitive function.

## 2. Material and Methods

### 2.1. Participants

The TLSA is a longitudinal survey with a nationally representative sample, conducted by the Health Promotion Administration (HPA), Ministry of Health and Welfare of Taiwan in collaboration with the Population Studies Center and the Institute of Gerontology of the University of Michigan in the United States in 1989 [18]. The TLSA was designed to study the impact of socioeconomic development on the physical and emotional well-being of Taiwanese older adults. The prospective investigation used a three-stage equal probability sampling design to obtain 4049 elderly participants (aged 60 years or more), with a survey response rate of 92% in the initial sample [18]. Trained interviewers conducted personal interviews through questionnaires at baseline and follow-up surveys in 1993, 1996, 1999, 2003, and 2007; the corresponding response rates were 92%, 91%, 89%, 90%, and 91%. The questionnaire included background information, family structure, health, use of medical services and hygiene habits, social support and exchange of support, employment history, leisure, activities, and general attitudes, economic status, and livelihood plans.

**Figure 1:**
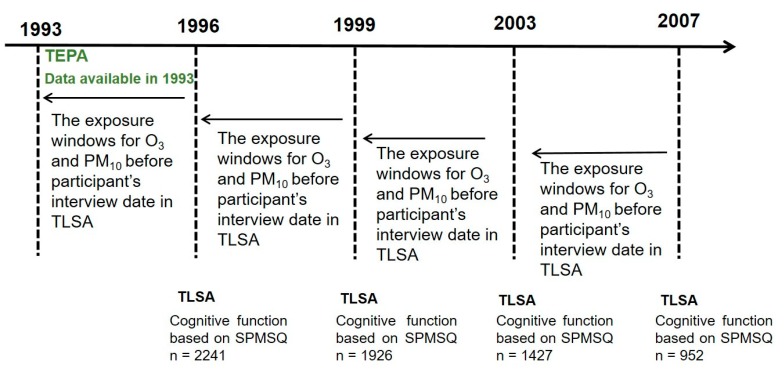
Flowchart of sample selection. Data source: Taiwan Longitudinal Study on Aging. Abbreviations: TEPA, Taiwan Environmental Protection Administration; TLSA, Taiwan Longitudinal Study on Aging; SPMSQ, Short Portable Mental Status Questionnaire.

Verbal consent was provided in all survey years prior to 2007, and written consent was obtained in 2007 by the HPA. In the present study, we used TLSA data from 1996 to 2007. Participants with Short Portable Mental Status Questionnaire (SPMSQ) scores that were less than 3 in 1993 (n = 72), incomplete in 1996 (n = 264), or with a stroke history in 1993 (n = 92) were excluded. Thus, there were 2241 elderly participants aged 65 years or more were included in 1996, 1926 in 1999, 1427 in 2003, and 952 in 2007 (Figure 1). This study was approved by the Institutional Review Board of the Tri-Service General Hospital (No.: 2-104-05-153). In order to increase sample sizes and capture the associations between time-dependent exposures and time-dependent outcomes, we used the repeated measurement study design suggested by the NSHAP study (National Social Life, Health and Aging project) [19].

### 2.2. Cognitive Function Measurement

Participants’ cognitive function was assessed using the five item form of the SPMSQ (SPMSQ), which was validated by a Chinese version of the Mini-Mental and has been used elsewhere [20,21,22,23]. The total Cronbach’s alpha value of SPMSQ was 0.63 for the TLSA sample. Participants were asked the following five questions: “What is today’s date (including month, day, and year)?”; “What is the day of the week?”; “What is your home address (or where are you)?”; and “How old are you?” They were also asked to subtract 3 from 20 for a total of four consecutive repetitions. One point was given for each correct answer, and the total score ranged from 0 to 5. We dichotomized the SPMSQ score and analyzed the risk for cognitive impairment because of the ceiling effect of SPMSQ; a SPMSQ score of <3 was used to identify individuals with moderate-to-severe cognitive impairment [21].

### 2.3. Exposure Assessment

**Figure 2:**
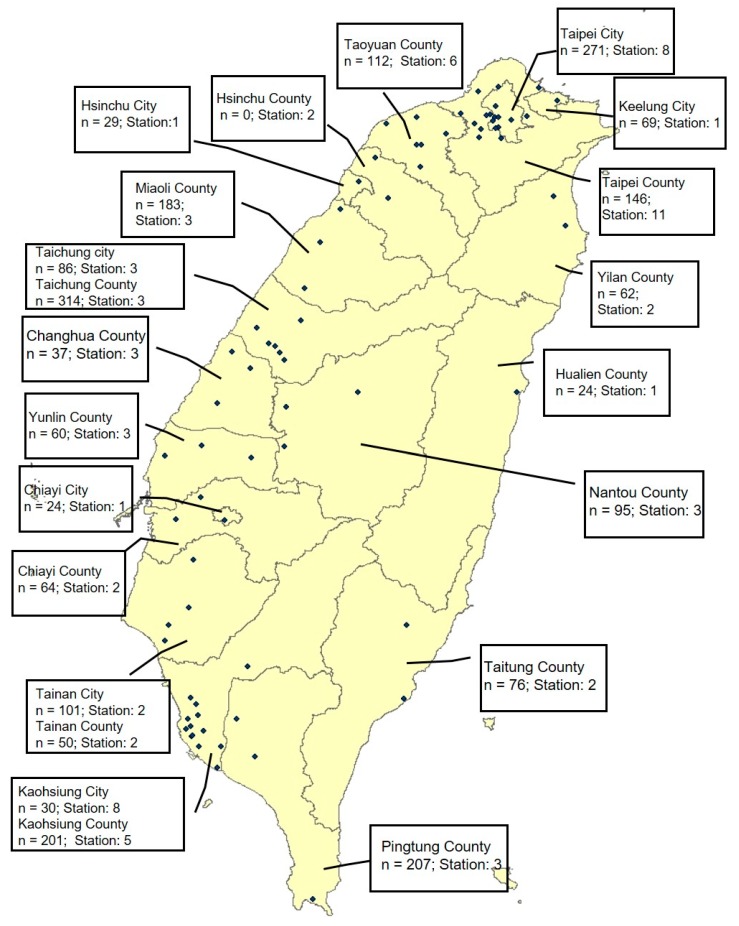
Distribution of study population (n = 2241) in 1996 and monitoring stations in each city/county in Taiwan.

We obtained hourly data of PM_10_ and O_3_ levels from 75 monitoring stations constructed by the Taiwan Environmental Protection Administration (TEPA) on Taiwan’s main island, which provided measurements continuously from 1993 to 2007 [24]. The methods used for measuring these pollutants were ultraviolet absorption for O_3_ and beta-gauge for PM_10_. These data were subjected to rigorous quality assurance and control procedures through independent projects. TEPA authorized an independent private sector organization to perform annual performance audits and regular performance checks of monitoring instruments. The daily average of air pollutants (PM_10_ and O_3_) obtained from each monitoring station was calculated for subsequent analyses. At least 75% of the 24 h values had to be available for the days included in this calculation. Subsequently, the daily average of air pollutants from monitoring stations within the same city or county was assigned to TLSA participants who lived in the same city or county [25]. If there was more than one monitoring station in a city/county, the data were averaged between them all for that city/county. We calculated the 7 day, 14 day, 21 day, 30 day, 60 day, 90 day, 180 day, 1 year, and 3 year averages prior to each participant’s interview date for each survey. Because the data for PM_2.5_ from all monitoring stations were only recorded in 2007, we did not include PM_2.5_ concentrations in exposure assessments in the present study. The distributions of study population and monitoring stations in each city/county in Taiwan are presented in Figure 2.

### 2.4. Covariates

Information on relevant covariates was collected through structured interviews. Data on demographic variables (e.g., age, sex, educational level, marital status, and current economic status) and lifestyle factors (e.g., smoking, alcohol consumption, and physical activity) were also obtained. Physical function was assessed based on participant’s ability to perform the instrumental activities of daily living (IADLs). IADLs consisted of shopping, managing money, riding a bus or train independently, doing heavy work around the house or yard, doing light housework, and using the telephone. The total scores ranged from 0 to 18, and higher scores indicated poorer functioning. Chronic diseases, including hypertension, diabetes, and heart disease were defined as whether participants reported having those diseases and whether they were diagnosed by a physician.

### 2.5. Statistical Analysis

Because of the longitudinal study design and multiple measurements per participant, we applied generalized linear mixed models fitted using the PROC GLIMMIX procedure to explore the relationship between cognitive function and exposure to ambient PM_10_ and/or O_3_, modeled as a binary outcome based on the SPMSQ score of <3 for moderate-to-severe cognitive impairment, as well as to account for random effects of repeated measurements for participants. We investigated PM_10_ and O_3_ exposure windows averaging from 7 days up to 3 years prior to the interview date of TLSA participants to assess the impact of acute and chronic PM_10_ and O_3_ exposure on cognitive function.

Covariates were selected based on whether they were associated with exposure, associated with outcomes, and not intermediate variables between exposure and outcome [26], as well as based on a 10% change-in-estimated criterion [27] including age, sex, education level, marital status, current economic status, smoking habits, alcohol consumption, physical activity, and IADL. To examine the interaction effects of exposure to PM_10_ and O_3_ on cognitive function, we categorized the exposure as greater or less than the average among participants. Additionally, we conducted sensitivity analyses only for participants who did not move, and the analyses were adjusted for hypertension and diabetes/or heart disease during the study period. All analyses were conducted using SAS statistical software (version 9.3; SAS Institute Inc., Cary, NC, USA), and the alpha level was set at 0.05.

## 3. Results

### 3.1. Study Population

Overall, participants were on average 73, 76, 78, and 82 years old in 1996, 1999, 2003, and 2007, respectively, and half were men (Table 1). Most participants were married, had primary and secondary school education, and had a self-reported financial status of fair or satisfied. More than half reported being physically active. Approximately one-fifth of participants were smokers and consumed alcohol in 1996. However, in 2007, only 12.0% of participants were smokers, and 18.8% consumed alcohol. Moreover, 26.6% and 47.2% of participants reported a history of hypertension in 1996 and 2007, respectively, and the corresponding percentages were 10.2% in 1996 and 14.2% in 2007 for diabetes. A total 15.7% and 25.2% of participants reported a history of heart disease in 1996 and 2007, respectively. Participants’ average SPMSQ scores were 4.49 in 1996 and 3.74 in 2007; IADL scores were 1.91 in 1996 and 4.22 in 2007. The percentage of current moderate-to-severe cognitive impairment increased from 5.20% in 1996 to 18.5% in 2007.

### 3.2. Distributions of PM_10_ and O_3_ Concentrations for Different Exposure Windows

**Figure 3:**
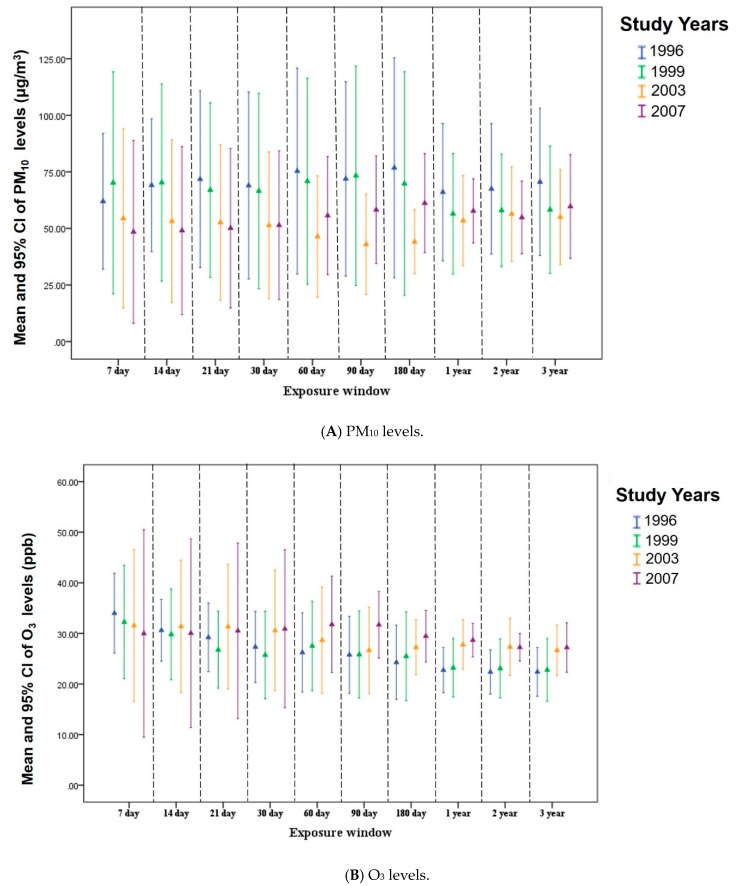
Mean and 95% Confidence Interval of PM_10_ (**A**) and O_3_ (**B**) concentrations in the 7 days up to 3 years (exposure windows) prior to each participant’s interview day from 1996 to 2007.

Figure 3 presents the distribution of PM_10_ and O_3_ concentrations for different exposure windows from 1993 to 2007. We found that the patterns of PM_10_ levels for the 7 day, 14 day, 21 day, 30 day, 60 day, 90 day, 180 day, 1 year, and 3 year exposure windows from 1996 to 2007 typically declined (*p* for trend <0.001) (Figure 3A). However, the patterns of O_3_ concentrations for the 21 day, 30 day, 60 day, 90 day, 180 day, 1 year, and 3 year exposure windows from 1996 to 2007 gradually increased (*p* for trend <0.001), except the patterns of O_3_ concentrations for the 7 day exposure window during the study period, which declined (*p* for trend <0.001) (Figure 3B).

### 3.3. Relations of Exposure to PM_10_ and O_3_ Levels and Cognitive Function

Table 2 presents the association of ambient PM_10_ and O_3_ in the previous 7, 14, 21, 30, 60, 90, and 180 days and in 1 and 3 years prior to each measure of cognitive function. After adjustment for covariates, a 10 μg/m^3^ increase in PM_10_ was significantly and positively associated with moderate-to-severe cognitive impairment for the 1 and 3 year exposure windows, with the largest increase in odds found for 3 year PM_10_ exposure (OR = 1.116; 95% CI: 1.041, 1.197). We further adjusted O_3_ concentrations, and the results were similar.

The models showed that a 10 μg/m^3^ increase in O_3_ was significantly and positively associated with moderate-to-severe cognitive impairment for the 30 day to 3 year exposure windows, with the largest increase in odds found for 1 year O_3_ exposure (OR = 1.974; 95% CI: 1.448, 2.691). We further adjusted PM_10_ concentrations, and the results were similar.

To examine the effect modification of long-term exposure to O_3_ and PM_10_ on cognitive function, we categorized the 1 year exposure window as greater or less than the average of these pollutants among participants (Table 3). We found that exposure to O_3_ >25 ppb and PM_10_ >60 µg/m^3^ may increase the odds of moderate-to-severe cognitive impairment (OR = 2.012, 95% CI: 1.473, 2.746) as compared with lower O_3_ and PM_10_ exposure, with a statistically significant interaction term (*p* < 0.001).

Additionally, when we restricted data analysis to only include participants who did not move and when we adjusted for hypertension and diabetes/or heart disease during the studying period, these results were still similar (data not shown).

## 4. Discussion

In our nationally representative sample of older adults in Taiwan, we observed statistically significantly positive associations of long-term PM_10_ and O_3_ exposure windows with moderate-to-severe cognitive impairment (e.g., OR = 1.083; 95% CI: 1.000, 1.074 for 1 year PM_10_ moving average; OR = 1.974; 95% CI: 1.448, 2.691 for 1 year O_3_ moving average). We also found that long-term co-exposure to PM_10_ and O_3_ may increase the risk of cognitive impairment. The results of this longitudinal follow-up study showed that exposure to air pollutants may impair cognitive function among community-dwelling, free-living older adults.

Few studies have examined the relationship of exposure to PM_10_ or O_3_ and cognitive function. Relevant studies have found an association of exposure to PM_10_ with decline in memory [28] and decline in general and test-specific performance abilities [14]. Tzivian et al. (2016) found that long-term (three years) exposure to air pollution (PM_10_) was positively associated with mild cognitive impairment in older adults [29]. On the other hand, we also found that long-term O_3_ exposure was associated with moderate-to-severe cognitive impairment in an elderly population. Chen and Schwaets (2009) reported an adverse association of exposure to O_3_ with cognitive performance in American adults [30]. Similarly, Cleary et al. investigated older adults aged over 60 years and found that increased levels of ozone were correlated with an increased rate of cognitive decline [31]. However, other studies have not observed any association between exposure to PM_10_ or O_3_ and cognitive decline [15,16,17]. These conflicting findings may be attributed to the varying sample sizes, different measurements of exposure, different measurement outcomes, varying demographic characteristics, different exposure windows, and different study designs.

In the two-pollutant models (Table 2), we observed that long-term exposure to PM_10_ and O_3_ were the independent predictors of cognitive impairment, although their magnitudes on cognitive impairment were different. To date, most studies have found that either PM or O_3_ exposure can be associated with cognitive function. Zhang et al. (2018) reported that long-term exposure to a high air pollution index (API) could impede cognitive performance among older adults in China [32]. However, the air pollution index (API) did not included O_3_ exposure in that study. As far as we know, this was the first study to provide the evidence that cumulate exposure to both PM and O_3_ could influence cognitive function in the elderly. Furthermore, previous studies have also indicated that exposure to both PM and O_3_ could increase the risks of Alzheimer’s disease [33] and mortality [34].

Our results demonstrated that long-term exposure to PM_10_ and O_3_ could be independent predictors of cognitive impairment in elderly adults, indicating that long-term exposure to PM_10_ and O_3_ could lead to cognitive impairment through different mechanisms. Animal studies have reported that PM may reach the brain via circulation or bypassing the multifaceted blood–brain barrier, through direct translocation through the olfactory bulb [35,36,37]. Experimental studies have shown that O_3_ exposure may affect neurodegenerative processes by increasing free radical levels, contributing to a state of elevated oxidative stress [9,38]. Furthermore, we observed an effect modification of long-term exposure to PM_10_ and O_3_ on cognitive impairment in our participants, indicating that long-term exposure to PM_10_ and O_3_ could accelerate cognitive impairment in older people. In vivo studies of acute and chronic low-level exposure to O_3_, PM, or PM–O_3_ mixtures have indicated neurotoxic effects in different animal models [6,7,8,38]. Previous studies have also suggested that exposure to a mixture of O_3_, PM, and other components may accelerate brain aging in dogs [9].

Our study had several limitations. In general, most of participants (about 99.9%) completed the questionnaire in person, however, a few participants (about 0.1%) with diplacusis, deafness, or mutism completed the questionnaire with the help of co-participants. Therefore, the information provided by participants could be inaccurate due to their cognitive impairment and the infrequent use of a co-participant to help fill out questionnaires.

First, cognitive function was assessed using the SPMSQ, which might restrict the results of our study. Although it is a valid and reliable instrument for identifying cognitive impairment, it is unlikely to identify those with subtle deficits [39]. However, the measure used in this study evaluated working memory and orientation, and deficits in the areas may reflect cognitive loss [22,40]. In addition, we did not have appropriate cognitive tests with which to measure individual-level decline over time, and this would be an aim for future studies.

Second, we could not collect the residential address of each participant; this could have led to measurement errors in estimated air pollutants as well as attenuated observed effects. Because we did not have data for PM_2.5_ before year 2007, it was difficult to assess the possible adverse health effects of exposure to PM_2.5_ on cognitive function among older adults. We could not rule out the possibility that these associations in the present study might have been partially due to the influence of PM_2.5_ if it correlated with PM_10_ and/or O_3_. Recently, a population-based cohort study using the National Health Insurance Database in Taiwan found that higher concentrations of O_3_ and PM were associated with increased risk of newly diagnosed Alzheimer’s disease (AD) [33], which indirectly supports our findings. Finally, the findings of the current study may not be generalizable to younger age groups.

The nationally representative sample of community-dwelling older adults in Taiwan was a major strength of our study. Our study was well-powered to detect meaningful associations and adjusted for potential confounding factors. Moreover, we showed an effect modification of exposure to PM_10_ and O_3_ on cognitive function, providing insight into the interplay of different air pollutants on cognitive impairment. We also considered multiple PM_10_ and O_3_ exposure windows and found that long-term exposure to PM_10_ and O_3_ may be the most biologically relevant exposure periods to cognitive function, compared with short-term exposure windows.

## 5. Conclusions

We provided evidence of positive associations between PM_10_ and O_3_ and cognitive impairment among a representative sample of Taiwanese older adults. Our findings suggest that exposure to ambient PM_10_ and O_3_ may increase the odds of cognitive impairment, and that interaction between pollutants may accelerate cognitive impairment.

## Figures and Tables

**Table 1:** Characteristics of TLSA study participants by survey year.

Variables	Year 1996 (n = 2241)	Year 1999 (n = 1926)	Year 2003 (n = 1427)	Year 2007 (n = 952)
Male, n (%)	1289 (57.5)	1088 (56.5)	797 (55.9)	498 (52.3)
Age, y, mean ± SD	73.62 ± 4.94	76.28 ± 4.73	78.79 ± 4.09	82.28 ± 3.76
Spouse, yes, n (%)	1362 (60.8)	1091 (56.6)	730 (51.2)	434 (45.6)
Personal education, n (%)				
Illiterate	805 (35.9)	672 (34.9)	463 (32.4)	311 (32.7)
Primary and secondary school	1162 (51.9)	1017 (52.8)	766 (53.7)	503 (52.8)
High school and above	274 (12.2)	237 (12.3)	198 (13.9)	138 (14.5)
Self-reported financial status, n (%)				
Very satisfied	178 (7.90)	140 (7.30)	83 (5.80)	52 (5.50)
Satisfied	757 (33.8)	628 (32.6)	594 (41.6)	396 (41.6)
Fair	965 (43.1)	775 (40.2)	479 (33.6)	340 (35.7)
Dissatisfied	278 (12.4)	289 (15.0)	207 (14.5)	122 (12.8)
Very dissatisfied	63 (2.80)	94 (4.90)	64 (4.50)	42 (4.40)
Physical activity, n (%)	913 (40.7)	1246 (64.7)	962 (67.4)	638 (67.0)
Smoking status, n (%)	586 (26.1)	436 (22.6)	247 (17.3)	114 (12.0)
Alcohol consumption, n (%)	421 (18.8)	410 (21.3)	283 (19.8)	179 (18.8)
Hypertension, n (%)	596 (26.6)	722 (37.5)	631 (44.2)	449 (47.2)
Diabetes, n (%)	228 (10.2)	294 (15.3)	243 (17.0)	135 (14.2)
Heart disease, n (%)	352 (15.7)	444 (23.1)	378 (26.5)	240 (25.2)
SPMSQ (0−5), (n (%), mean ± SD)	4.49 ± 0.89	4.45 ± 0.91	4.20 ± 1.03	3.74 ± 1.32
≥3	2124 (94.8)	1817 (94.3)	1313 (92.0)	776 (81.5)
<3	117 (5.20)	109 (5.7)	114 (8.00)	179 (18.5)
IADL (0−18), mean ± SD	1.91 ± 3.62	2.37 ± 3.95	3.31 ± 4.54	4.22 ± 5.06

**Table 2:** ORs (95% CIs) for moderate-to-severe cognitive impairment per 10 µg/m^3^ PM_10_ and per 10 ppb O_3_ (n = 6546).

**PM_10_ (µg/m^3^)** **Moving Averages**	**SPMSQ < 3**	***p*-Value**	**SPMSQ < 3**	***p*-Value**
	**OR (95%CI) ^AD^**		**OR (95%CI) ^BD^**	
7 days	1.020 (0.980, 1.062)	0.390	1.030 (0.990, 1.083)	0.218
14 days	1.010 (0.961, 1.062)	0.691	1.000 (0.961, 1.062)	0.731
21days	1.020 (0.980, 1.073)	0.331	1.010 (0.970, 1.062)	0.526
30 days	1.030 (0.990, 1.083)	0.212	1.020 (0.980, 1.073)	0.337
60 days	1.030 (0.990, 1.083)	0.173	1.020 (0.980, 1.073)	0.331
90 days	1.030 (1.000, 1.083)	0.102	1.030 (0.990, 1.073)	0.218
180 days	1.041 (1.000, 1.094)	0.032	1.041 (1.000, 1.083)	0.063
1 year	1.083 (1.000, 1.174)	0.035	1.083 (1.000, 1.174)	0.039
3 years	1.116 (1.041, 1.197)	0.001	1.094 (1.020, 1.174)	0.007
**O_3_ (ppb)** **Moving Averages**	**SPMSQ < 3**	***p*-Value**	**SPMSQ < 3**	***p*-Value**
	**OR (95%CI) ^AD^**		**OR (95%CI) ^CD^**	
7 days	0.961 (0.827, 1.094)	0.510	0.923 (0.787, 1.062)	0.272
14 days	1.010 (0.869, 1.197)	0.828	1.000 (0.852, 1.197)	0.926
21days	1.150 (0.980, 1.363)	0.101	1.127 (0.961, 1.350)	0.143
30 days	1.221 (1.030, 1.448)	0.017	1.209 (1.020, 1.433)	0.024
60 days	1.419 (1.162, 1.733)	<0.001	1.405 (1.150, 1.716)	<0.001
90 days	1.682 (1.350, 2.096)	<0.001	1.649 (1.323, 2.054)	<0.001
180 days	1.751 (1.350, 2.248)	<0.001	1.716 (1.323, 2.203)	<0.001
1 year	1.974 (1.448, 2.691)	<0.001	1.954 (1.448, 2.664)	<0.001
3 years	1.954 (1.433, 2.664)	<0.001	1.878 (1.363, 2.560)	<0.001

**^A^** Model was adjusted for age, sex, personal education, marital status, self-reported financial status, smoking, alcohol consumption, physical activity, and IADL; **^B^** Model was adjusted for age, sex, personal education, marital status, self-reported financial status, smoking, alcohol consumption, physical activity, IADL, and O_3_; **^C^** Model was adjusted for age, sex, personal education, marital status, self-reported financial status, smoking, alcohol consumption, physical activity, IADL, and PM_10_. **^D^** The SPMSQ score is ≥3 as a reference group.

**Table 3:** Combined effects of long-term exposure to O_3_ and PM_10_ on moderate-to-severe cognitive impairment (n = 6546).

Variables	n	SPMSQ < 3		*p*-Value ^B^
		OR ^A,C^	95%CI	
O_3_ and PM_10_ 1 year moving averages				<0.001
O_3_ ≤ 25 (ppb) and PM_10_ ≤ 60 (µg/m^3^)	2099	Reference		
O_3_ ≤ 25 (ppb) and PM_10_ > 60 (µg/m^3^)	1568	1.510	(1.087, 2.098)	
O_3_ > 25 (ppb) and PM_10_ ≤ 60 (µg/m^3^)	1654	1.852	(1.381, 2.479)	
O_3_ > 25 (ppb) and PM_10_ > 60 (µg/m^3^)	1225	2.012	(1.473, 2.746)	

**^A^** Model was adjusted for age, sex, personal education, marital status, self-reported financial status, smoking, alcohol consumption, physical activity, and IADL; **^B^** *p* for interaction term; **^C^** The SPMSQ score is ≥3 as a reference group.
